# Effectiveness of early initiation of idursulfase in infants and very young children with mucopolysaccharidosis II: a retrospective chart review in siblings

**DOI:** 10.1016/j.ymgmr.2026.101354

**Published:** 2026-09-25

**Authors:** Barbara K. Burton, Bernd Schweikert, Hernàn Amartino, Maira Sharipova, Jaime Lopez-Valdez, Deniz Kor, Huseyin Bilgin, Walla Al-Hertani, Ana Maria Martins, Dimitrios Zafeiriou, Nathalie Guffon, Olivia Okoli, Diana Sanchez-Hernandez, Jennifer Audi, Jaco Botha, Csaba Siffel, Olulade Ayodele

**Affiliations:** a Ann & Robert H. Lurie Children's Hospital of Chicago, Northwestern University, 225 E, Chicago Ave., Chicago, IL 60611, United States of America; b ICON plc, 26 Heinrich-Herz-Stra., 63225 Langen, Germany; c Hospital Universitario Austral, Juan Domingo Peron 1500, B1629 Pilar Centro, Buenos Aires, Argentina; d Scientific Center of Pediatrics and Pediatric Surgery JSC, Al-Farabi Avenue 146, Almaty 050060, Kazakhstan; e Centenario Hospital Miguel Hidalgo, Avenida Gómez Morín S/N, La Estación La Alameda, CP 20259, Aguascalientes, Mexico; f Cukurova University, Medical Faculty, Department of Pediatric Metabolism and Nutrition, 01330 Saricam, Adana, Turkey; g Department of Pediatrics, Division of Metabolism, Diyarbakir Children's Hospital, Fırat Blv. No: 12, 21070 Diyarbakir, Turkey; h Division of Genetics and Genomics, Boston Children's Hospital, Harvard Medical School, 300 Longwood Ave., Boston, MA 02115, United States of America; i Division of Metabolic Disorders, Children's Hospital of Orange County (CHOC®), Rady Children's Health, 1201 W. La Veta Ave., Orange, CA 92868, United States of America; j Universidade Federal de São Paulo, Centro de Referência em Erros Inatos do Metabolismo, Rua Botucatu, 862, SP 04023-062, Brazil; k 1st Department of Pediatrics, Medical School, Aristotle University, Hippokratio General Hospital, Konstantinoupoleos 49, Thessaloniki 546 42, Greece; l Reference Center for Inherited Metabolic Diseases, Hospices Civils de Lyon, Hôpital Femme Mère Enfant, 59 Boulevard Pinel, 69500 Bron, Lyon, France; m ICON plc, 500 South Oak Way, Green Park, Reading RG2 6AD, UK; n ICON plc, Suite #206, 5420 North Service Road, Burlington, ON L7L 6C7, Canada; o Takeda Pharmaceuticals International AG, Thurgauerstrasse 130, 8152 Glattpark-Opfikon, Zurich, Switzerland; p College of Allied Health Sciences, Augusta University, 1120 15th St., Augusta, GA 30912, United States of America; q Takeda Development Center Americas, Inc., 500 Kendall St., Cambridge, MA 02142-1108, United States of America

**Keywords:** Early treatment, Enzyme replacement therapy, Hunter syndrome, Idursulfase, Lysosomal storage diseases, Mucopolysaccharidosis II

## Abstract

**Introduction:**

Enzyme replacement therapy (ERT) with intravenous idursulfase is the standard of care for patients with mucopolysaccharidosis II (MPS II; Hunter syndrome). Data are limited for children aged ≤12 months. This retrospective study evaluated outcomes with early ERT.

**Methods:**

Male siblings with MPS II treated with idursulfase for >2 years were categorized into five cohorts by age at treatment initiation. The primary objective compared Cohort 1 (ERT initiation aged ≤12 months) with Cohort 2 (ERT initiation at an age ≥ 12 months older). Clinical outcomes were compared using Kaplan–Meier and Cox proportional models, and generalized linear mixed models (GLMMs) for variables with adequate data.

**Results:**

Overall, 38 patients (19 sibling pairs) were included; the primary analysis included 15 per cohort. Median (10th–90th percentile) age at idursulfase initiation was 3.0 (1.0–11.0) and 44.0 (28.0–84.0) months and mean (standard deviation) follow-up was 69.7 (42.5) and 130.6 (46.8) months in Cohorts 1 and 2, respectively. First events from birth of abnormal liver size, joint stiffness, carpal tunnel syndrome, and enlarged tonsils and adenoids occurred less frequently in Cohort 1 than 2; first events of surgery or cardiac valve disease occurred more frequently in Cohort 1 than 2. GLMM analyses showed that, compared with their older siblings, patients who initiated ERT aged ≤12 months had better clinical outcomes for height-for-age *Z*-score, liver, cardiac, and musculoskeletal manifestations.

**Discussion:**

Early ERT may improve clinical outcomes in MPS II, supporting the known efficacy of idursulfase and adding to the limited evidence for early ERT.

## Highlights

• Mucopolysaccharidosis II (MPS II) awareness enables earlier diagnosis and treatment.
• Benefits of early enzyme replacement therapy may be understood by studying siblings.
• Studying siblings with MPS II may help to address unmeasured confounding factors.
• Data suggested that better outcomes may be achieved with early treatment initiation.
• Observations add to limited data supporting idursulfase in patients aged ≤12 months.

## Graphical abstract

**Figure:**
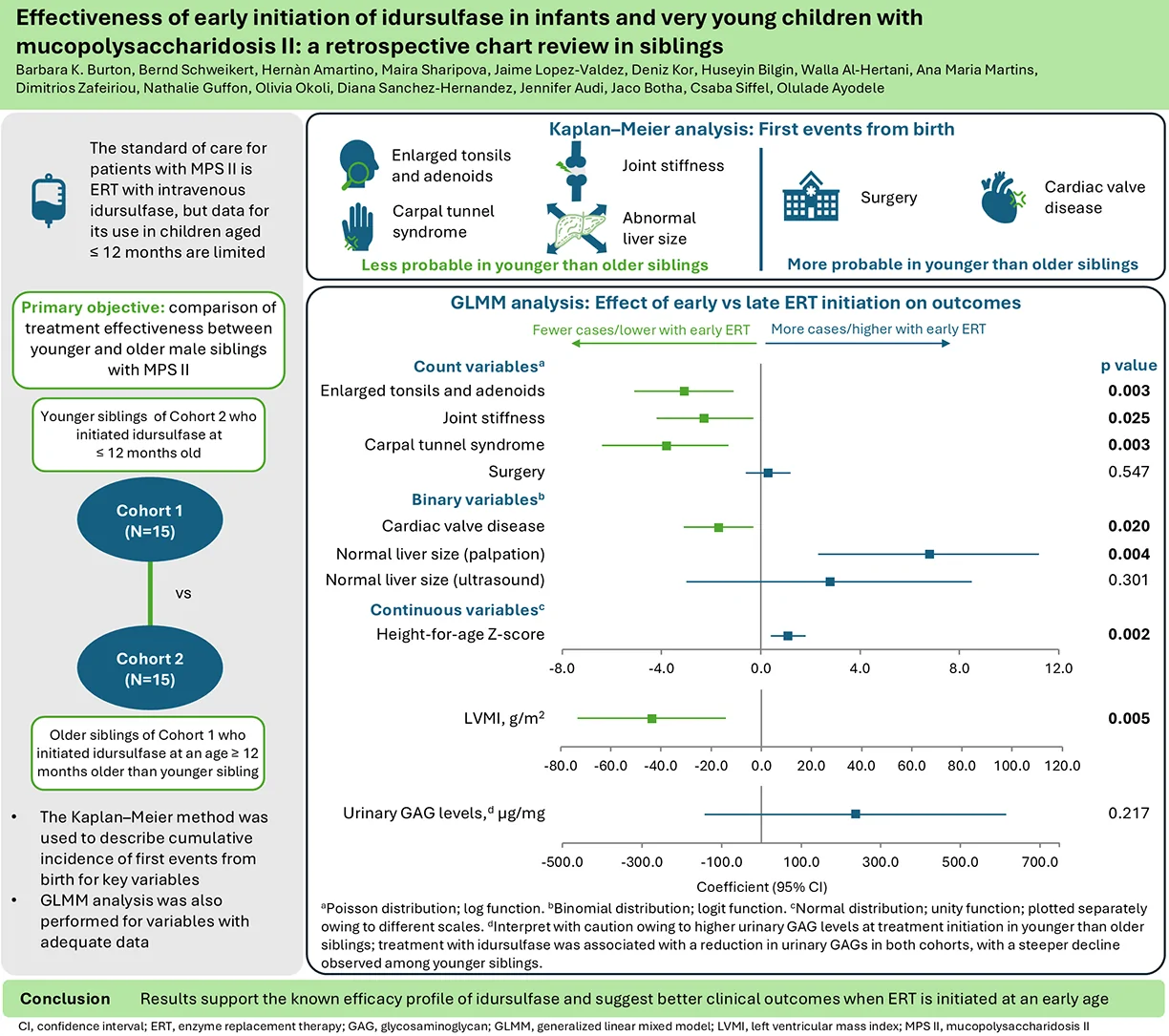

## 1 Introduction

Mucopolysaccharidosis II (MPS II; Hunter syndrome; OMIM 309900) is a rare, life-limiting, X-linked lysosomal disease caused by deficient iduronate-2-sulfatase activity due to pathogenic variants of the *IDS* gene [1]. This enzyme deficiency results in the accumulation of partially degraded glycosaminoglycans (GAGs) throughout the body, resulting in multisystemic disease and reduced life expectancy [1], [2]. Patients with MPS II do not typically exhibit signs or symptoms until 2–4 years of age [2], and clinical heterogeneity in the number of clinical signs and symptoms of disease and their time of presentation makes diagnosis challenging [3]. As a result, many patients experience a diagnostic delay of 2–4.5 years [4], [5], although other studies have reported this to be much higher [6].

The standard of care for MPS II is enzyme replacement therapy (ERT) with intravenous (IV) idursulfase (Elaprase®), which has been shown to improve pulmonary function, stabilize left ventricular mass index (LVMI), increase 6-min walk test (6MWT) distance, decrease liver and spleen volumes [7], and prolong survival compared with no treatment [1]. ERT is not curative; the goal of treatment is to slow or stop the progression of disease before organs become irreversibly damaged [2], [3], [8]. It has been suggested that early treatment with ERT provides better clinical outcomes [8], however, data supporting the safety or effectiveness of early ERT are limited. The pivotal trial of idursulfase (NCT00069641) excluded patients under the age of 5 years owing to the nature of the primary endpoints, which required participants to be able to perform pulmonary function tests and the 6MWT [9]. A subsequent open-label study that measured clinical efficacy of ERT by changes in liver and spleen sizes (NCT00607386) was able to demonstrate the clinical benefits of weekly 0.5 mg/kg IV idursulfase in children aged 1.4–7.5 years (mean age of 4.0 years). Adverse events were similar to those reported in the pivotal trial [10]. In an observational study of patients who initiated idursulfase before the age of 6 years (mean age at treatment initiation: 3.6 years), no new safety concerns were identified [2]. Data to support the use of idursulfase in patients with lysosomal diseases who were younger than 18 months of age are limited to case reports [11], [12] and data for patients with MPS II who initiated ERT aged younger than 12 months are limited to a case series of eight patients in whom age at treatment initiation ranged from 10 days to 6.5 months [13]. A previous case study of a patient with neuronopathic MPS II reported favorable outcomes with a multimodal therapeutic approach comprising IV idursulfase (initiated at 2 months of age), hematopoietic stem cell transplantation (HSCT; at 23 months of age), and intracerebroventricular idursulfase (from 10 months of age) [14].

To further explore the real-world use and effectiveness of ERT in very young children with MPS II, we conducted a retrospective study to evaluate outcomes in patients who initiated IV idursulfase aged 12 months or younger compared with their older siblings who initiated treatment at an age at least 12 months older than their younger sibling.

## 2 Methods

### 2.1 Objectives

The primary objective was to evaluate the real-world effectiveness of ERT with IV idursulfase in very young (aged 12 months or younger) children with MPS II compared with an older sibling who initiated treatment at an age at least 12 months older. Exploratory objectives included assessing the impact and effectiveness of early treatment with idursulfase in patients who initiated treatment aged 12 months or younger, or 20 months or younger, compared with their older siblings who initiated treatment at 36 months of age or older, or at an age at least 12 months older than the age of their younger sibling at treatment initiation, respectively. Study cohorts are shown in Fig. 1A.

**Fig. 1:**
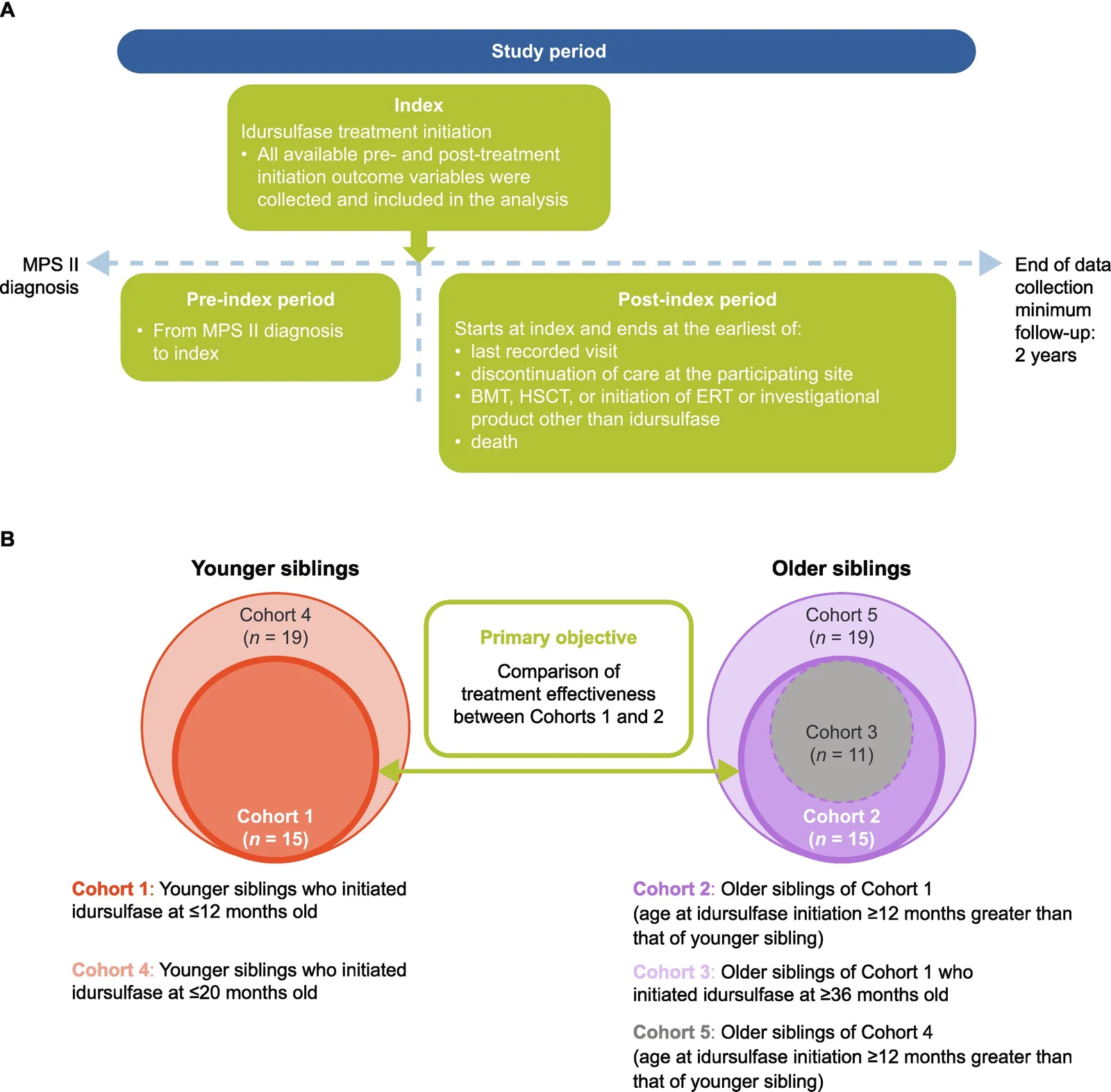
Study design and cohorts. ERT, enzyme replacement therapy; HSCT, hematopoietic stem cell transplantation; MPS II, mucopolysaccharidosis II.

Safety was not an objective of the study; however, documented adverse events in patients' medical charts were captured at data collection.

### 2.2 Study design

In this global, multicenter, noninterventional retrospective chart review, patient data were abstracted between March 4, 2022 and February 8, 2023. Sibling pairs with MPS II who initiated ERT with idursulfase at different time points in their lives were included. The study design is shown in Fig. 1B. Index was defined as the patient's age in months at idursulfase initiation. The pre-index period was defined by the difference in months between the patient's age at index and their age at diagnosis of MPS II. The follow-up period was defined by the difference in months between the patient's age at index and their age at whichever came first of: last recorded visit, discontinuation of care at the participating site, HSCT, initiation of an ERT or investigational product other than idursulfase, or death. The minimum follow-up time was 2 years.

### 2.3 Data source

Data were obtained from existing medical charts at 14 participating sites across Europe, Latin America, North America, Turkey, and Kazakhstan. Owing to the political climate at the time of the study, one site in Russia was not initiated in the study. A medical insights questionnaire administered by Takeda Global Medical Affairs was used to locate sibling pairs and determine whether they fit the inclusion criteria. Waivers of consent (and patient consent, where applicable) were granted by the Institutional Review Board and/or Institutional Ethics Committee for each site participating in the study. All relevant data were abstracted and entered by the site principal investigator or delegated staff into an electronic case report form (eCRF) that was developed to capture patient characteristics and outcomes. The eCRF was programmed by the Clinical Research Organization (CRO; ICON Clinical Research Ltd) and was compliant with FDA 21 CRF Part 11 requirements. The management of data collection by the CRO was funded by Takeda Pharmaceuticals. Steps were taken to minimize the amount of missing data and optimize the integrity of the analysis by allowing for an ‘unknown’ or ‘not available’ option for most questions in the eCRF.

### 2.4 Study population

Sibling pairs were eligible for inclusion if they both met the following criteria: male; genetic or biochemical diagnosis of MPS II; had received idursulfase treatment for MPS II for at least 2 years, with the younger sibling starting treatment aged 20 months or younger and the older sibling starting treatment at an age at least 12 months older than the age their younger sibling started treatment. Patients were excluded from the analysis if they met the following criteria: received treatment for MPS II with an ERT or investigational product other than idursulfase (intrathecal or IV) within 2 years of initiating treatment with idursulfase; underwent HSCT within 2 years of initiating treatment with idursulfase.

Patients were placed into five cohorts and sub-cohorts for the purposes of the analyses (Fig. 1A). Cohort 1 included younger siblings who initiated treatment with idursulfase aged 12 months or younger. Cohort 2 were older siblings of Cohort 1 who initiated treatment with idursulfase at an age at least 12 months older than that of their younger sibling. Cohort 3 was a subset of Cohort 2, including patients who initiated idursulfase aged 36 months or older. Cohort 4 were younger siblings who initiated treatment with idursulfase at 20 months of age or younger, and included all patients from Cohort 1. Cohort 5 were older siblings of Cohort 4, and therefore included all patients from Cohorts 2 and 3. Patients in Cohort 5 initiated treatment with idursulfase at an age at least 12 months older than that of their younger sibling.

For the primary objective, comparisons were made between Cohort 1 and Cohort 2. For the exploratory objectives, comparisons were made between Cohort 1 and Cohort 3, and between Cohort 4 and Cohort 5.

### 2.5 Variables and outcomes

Patient characteristics were abstracted from medical charts, depending on availability. Cognitive impairment was clinician-reported and was used primarily to characterize MPS II severity (neuronopathic or attenuated) and serve as a baseline descriptor for sibling comparisons. Given that intravenous idursulfase is not expected to cross the blood–brain barrier [2], cognitive impairment was not considered a primary efficacy outcome of ERT in this study. The following clinical outcomes were assessed during the post-index period: urinary GAG levels, signs and symptoms by organ system (presence of symptoms, age at onset, frequency of recurrence, and severity), and healthcare resource utilization (surgery). A full list of study variables and outcomes is presented in Supplementary Table S1.

### 2.6 Statistical analysis

A statistical analysis plan was developed and finalized before any data analyses were performed, and defined all analytic populations and subpopulations. All analyses were purely descriptive because no predefined hypothesis was tested, and were restricted to the subset of patients for whom data were available.

For continuous variables, data were presented as the number of nonmissing observations, mean, standard deviation (SD), median, and 10th and 90th percentiles (P10 and P90). Frequencies and percentages were presented for categorical data.

The cumulative incidence of ‘first events’ from birth by cohort were estimated in a time-to-event analysis using the Kaplan–Meier method. Hazard ratios (HRs) and 95% confidence intervals (CIs) for cohort variables were calculated from a Cox proportional model, including the cohort as a single covariate.

A generalized linear mixed model (GLMM) was performed for a selected number of binary and continuous variables for cohorts with an adequate number of patients. The model considered the correlation between the younger and older sibling and the correlation in observations within the same patient over time. The CIs and *p* values calculated in the GLMM analysis should not be interpreted in the strict sense of confirmatory testing but rather as an indicator of precision and uncertainty. However, stronger correlations (*p* < 0.05) may be considered indicative of a stronger effect related to treatment initiation at a younger age. The statistical analyses for this paper were generated using SAS software, Version 9.4 (SAS Studio Release 3.82, Enterprise Edition).

## 3 Results

### 3.1 Patient characteristics

In total, 38 patients (19 sibling pairs) were included in the study and grouped into one or more of the five cohorts (Cohort 1, *n* = 15; Cohort 2, *n* = 15; Cohort 3, *n* = 11; Cohort 4, *n* = 19; Cohort 5, *n* = 19). For the primary objective (Cohort 1 vs Cohort 2), 30 patients (15 sibling pairs) were included in the analyses.

Baseline demographics and clinical and treatment characteristics for the primary study objective are summarized in Table 1. Data for cohorts addressing the exploratory objectives are summarized in Supplementary Table S2. For Cohorts 1 and 2, median (P10–P90) age at MPS II diagnosis was 2 (0–9) months and 36 (21–81) months, and median (P10–P90) age at idursulfase initiation was 3 (1−11) and 44 (28–84) months, respectively; all patients were male. All patients initiated idursulfase on a once-weekly regimen; some had one or more modifications of dose and/or frequency at different times during follow-up. Younger siblings received slightly higher doses of idursulfase than older siblings (mean [SD] 0.6 [0.3] mg/kg in Cohorts 1 and 4 compared with 0.5 [0.1] mg/kg in Cohorts 2 and 5, and 0.5 [0.0] mg/kg in Cohort 3). Adherence (defined as receiving >80% of infusions) was similar between Cohorts 1 and 2 (86.7% of patients each). The most common type of pathogenic *IDS* variant was missense mutation, which was present in over 47% of patients in each cohort.

**Table 1:** Patient characteristics (primary analysis: Cohort 1 vs Cohort 2).

Characteristic	Cohort 1	Cohort 2
	*n* = 15	*n* = 15
Median (P10–P90) age at MPS II diagnosis, months	2 (0–9)	36 (21–81)
Median (P10–P90) age at idursulfase initiation, months	3 (1–11)	44 (28–84)
Median (P10–P90) follow-up duration from index, months	49 (34–142)	96 (60–143)
Cognitive impairment at time of data collection, *n* (%)	6 (40.0)	12 (80.0)
Diagnosed by clinical judgment, *n* (%)	4 (26.7)	8 (53.3)
Diagnosed by formal testing, *n* (%)	2 (13.3)	4 (26.7)
Deceased, *n* (%)	1 (6.7)	6 (40.0)
Median (P10–P90) age at death, months	43 (−)	150.5 (120–205)
Cause of death, *n* (%)		
Acute respiratory insufficiency	0	1 (6.7)
Central apnea	0	1 (6.7)
Mechanical complication of tracheostomy	0	1 (6.7)
MPS II disease evolution	0	1 (6.7)
Pneumonia	1 (6.7)	2 (13.3)
Mean (SD) idursulfase dose at initiation, mg/kg	0.6 (0.3)	0.5 (0.1)
Adherence to treatment,^a^*n* (%)	13 (86.7)	13 (86.7)
Discontinued treatment, *n* (%)	2 (13.3)	4 (26.7)
Median (P10–P90) age at discontinuation, months	31 (26–36)	114 (52–181)
Reason for discontinuation, *n* (%)		
Enrolled in clinical trial	2 (13.3)	2 (13.3)
Death	0	1 (6.7)
Neurological disease	0	1 (6.7)
Continued treatment, *n* (%)	13 (86.7)	11 (73.3)
Treatment continuation unknown, *n* (%)	0	0
Pathogenic *IDS* variant, *n* (%)		
Missense	8 (53.3)	8 (53.3)
Splicing^b^, ^c^	4 (26.7)	3 (20.0)
Nonsense	1 (6.7)	1 (6.7)
Frameshift	1 (6.7)	1 (6.7)
Deletion	1 (6.7)	1 (6.7)
Recombination	0	0
Not reported	0	1 (6.7)

*IDS*, *iduronate-2-sulfatase*; MPS II, mucopolysaccharidosis II; P10, 10th percentile; P90, 90th percentile; SD, standard deviation.

a Defined as receiving >80% of infusions.

b One pair of siblings had a missense mutation (single nucleotide substitution) that disrupted splicing; they were included in the splicing category.

c The discrepancy in counts between younger and older siblings is attributable to one older sibling whose genotype was not reported.

In all primary and exploratory analysis cohorts, cognitive impairment was more prevalent among older siblings (Cohort 2, 80.0%; Cohort 3, 90.9%; Cohort 5, 84.2%) than younger siblings (Cohort 1, 40.0%; Cohort 4, 47.4%) and was typically diagnosed by clinical judgment. The last known vital status for most patients in both primary and exploratory analyses was recorded as ‘alive’ (*n* = 30 patients overall [78.9%]). Among patients who were deceased, the most common cause of death was pneumonia (range 5.3–18.2% patients across all cohorts).

### 3.2 First events of clinical outcomes, signs, and symptoms

Time-to-event analyses showed that HRs for a first event from birth of cardiac valve disease were above 1 in younger siblings in Cohort 1 (HR 1.08; 95% CI: 0.41, 2.84; Fig. 2A) and Cohort 4 (HR 1.25; 95% CI: 0.55, 2.86; Supplementary Fig. S2A) versus older siblings in Cohort 2 and Cohort 5, respectively. For younger siblings in Cohort 1 versus older siblings in Cohort 3, HRs were approximately 1 for a first event from birth of cardiac valve disease (HR 1.00; 95% CI: 0.32, 3.09; Supplementary Fig. S1A).

**Fig. 2:**
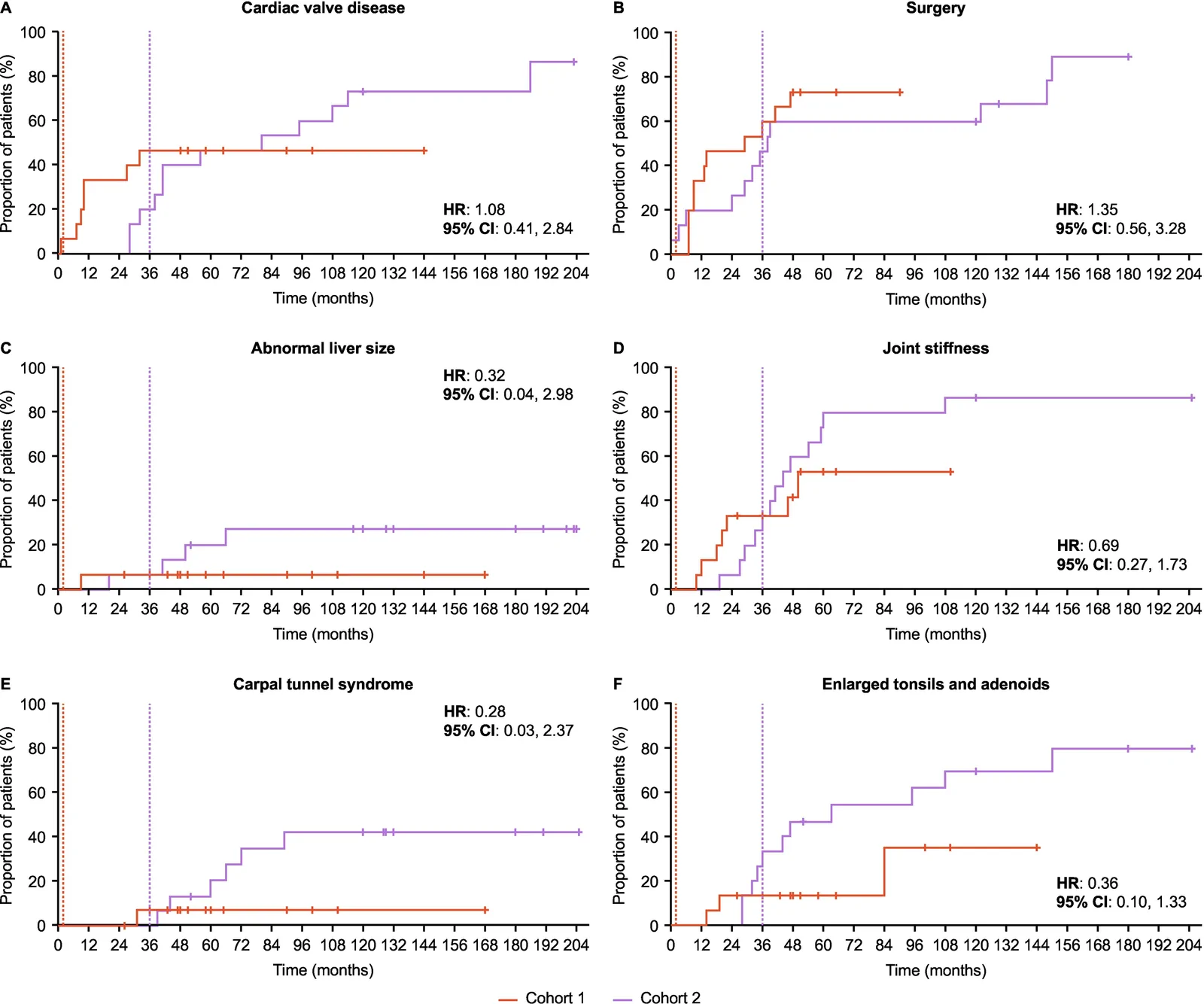
Cumulative incidence of first event from birth (primary analysis Cohort 1 vs Cohort 2). Dashed line shows median age at diagnosis. CI, confidence interval; HR, hazard ratio.

For surgery related to MPS II, HRs were above 1 for younger versus older siblings in both the primary (Cohort 1 vs Cohort 2: HR 1.35; 95% CI: 0.56, 3.28; Fig. 2B) and exploratory analyses (Cohort 1 vs Cohort 3: HR 1.68; 95% CI: 0.58, 4.88; Supplementary Fig. S1B, and Cohort 4 vs Cohort 5: HR 1.03; 95% CI: 0.47, 2.27; Supplementary Fig. S2B).

In the primary analysis, HRs were below 1 for younger siblings versus older siblings to experience a first event from birth for abnormal liver size (HR 0.32; 95% CI: 0.04, 2.98), joint stiffness (HR 0.69; 95% CI: 0.27, 1.73), carpal tunnel syndrome (HR 0.28; 95% CI: 0.03, 2.37), and enlarged tonsils and adenoids (HR 0.36; 95% CI: 0.10, 1.33) (Fig. 2C–F). Similar results were observed in the exploratory analyses (Supplementary Fig. S1 and S2) for abnormal liver size (Cohort 1 vs Cohort 3: HR 0.44; 95% CI: 0.05, 4.26; Cohort 4 vs Cohort 5: HR 0.43; 95% CI: 0.09, 2.16), joint stiffness (Cohort 1 vs Cohort 3: HR 0.74; 95% CI: 0.25, 2.22; Cohort 4 vs Cohort 5: HR 0.54; 95% CI: 0.23, 1.28), carpal tunnel syndrome (Cohort 1 vs Cohort 3: HR 0.28; 95% CI: 0.03, 2.40; Cohort 4 vs Cohort 5: HR 0.22; 95% CI: 0.03, 1.79), and enlarged tonsils and adenoids (Cohort 1 vs Cohort 3: HR 0.40; 95% CI: 0.11, 1.51; Cohort 4 vs Cohort 5: HR 0.37; 95% CI: 0.12, 1.16).

Younger siblings in the primary analysis exhibited a lower plateau in the cumulative incidence of first events than older siblings (Fig. 2). The same was true in the exploratory analyses, with the exception of incidence of first event of abnormal liver size, for which a higher plateau was observed in younger siblings of Cohort 4 (compared with their older siblings in Cohort 5; Supplementary Fig. S2D). However, follow-up times for younger siblings were typically shorter than for older siblings because they started treatment more recently than their older siblings, resulting in fewer total years of follow-up.

### 3.3 Post-treatment clinical outcomes

At index, mean (SD) urinary GAG levels were reported as 1121.0 (754.3) μg/mg in Cohort 1 (median [P10–P90] age at initiation 3.0 [1.0–11.0] months) and 380.3 (199.6) μg/mg in Cohort 2 (median [P10–P90] age at initiation 44.0 [28.0–84.0] months). However, shortly after treatment initiation (6–12 months), the decline in urinary GAG levels from baseline was larger among younger versus older siblings (Fig. 3A; Supplementary Table S3). Similar results were observed in exploratory analyses between Cohort 1 and Cohort 3 (Supplementary Table S4) and Cohort 4 and Cohort 5 (Supplementary Table S5).

**Fig. 3:**
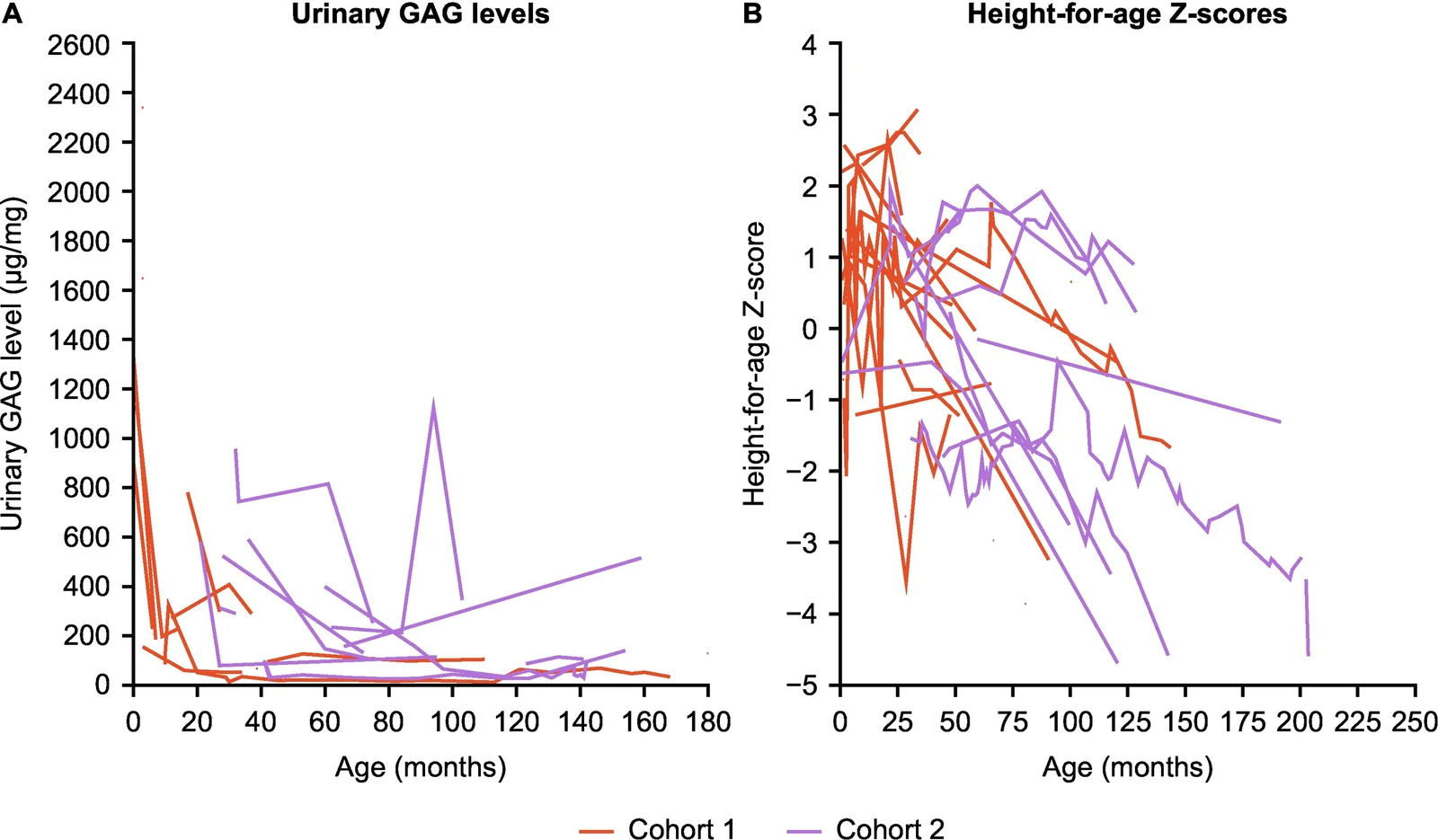
Clinical outcomes for continuous variables (primary analysis Cohort 1 vs Cohort 2). GAG, glycosaminoglycan.

Height-for-age *Z*-score was similar between younger and older sibling cohorts at index. After the initiation of idursulfase, all cohorts experienced fluctuations in height-for-age *Z*-score (Fig. 3B, Supplementary Tables S4 and S5).

It was not possible to conclusively interpret the effect of treatment on LVMI owing to the scarcity of data in each of the cohorts, although available data suggest that LVMI decreased following idursulfase initiation (Supplementary Tables S3–S5).

Younger siblings tended to have longer lifespans than older siblings (Cohort 1 vs Cohort 2: HR 0.33; 95% CI: 0.04, 2.89; Cohort 1 vs Cohort 3: HR 0.35; 95% CI: 0.04, 3.05; Cohort 4 vs Cohort 5: HR 0.28; 95% CI: 0.03, 2.39); however, this should be interpreted with caution owing to differences in follow-up times (Supplementary Fig. S3).

At index, the presence of cardiac valve disease was higher in Cohort 2 (*n* = 6, 40.0%) compared with Cohort 1 (*n* = 3, 20.0%), and in Cohort 3 (*n* = 4, 36.4%) compared with Cohort 1 (*n* = 2, 18.2%) (Table 2 and Supplementary Table S6). Cohort 4 and Cohort 5 showed a similar prevalence of cardiac valve disease at index (*n* = 6, 31.6% and *n* = 7, 36.8%, respectively; Supplementary Table S7). In all cohorts, where data were available, a general decrease in the presence of cardiac valve disease was observed after the initiation of idursulfase (Table 2, Supplementary Fig. S4A and Supplementary Tables S6 and S7).

**Table 2:** Clinical outcomes for cardiac valve disease, surgery, and normal liver size (primary analysis: Cohort 1 vs Cohort 2).

	Cardiac valve disease		Surgery		Normal liver size (ultrasound)		Normal liver size (palpation)	
Time of outcome measurement	Cohort 1 *n* = 15	Cohort 2 *n* = 15	Cohort 1 *n* = 15	Cohort 2 *n* = 15	Cohort 1 *n* = 15	Cohort 2 *n* = 15	Cohort 1 *n* = 15	Cohort 2 *n* = 15
Index, *n* (missing) Yes (%)	7 (8) 3 (20.0)	11 (4) 6 (40.0)	–	4 (11) 4 (26.7)	2 (13) 1 (6.7)	5 (10) 2 (13.3)	7 (8) 4 (26.7)	8 (7) 0
12 months post initiation, *n* (missing) Yes (%)	3 (12) 0	2 (13) 2 (13.3)	1 (14) 1 (6.7)	1 (14) 1 (6.7)	2 (13) 1 (6.7)	–	2 (13) 1 (6.7)	2 (13) 0
24 months post initiation, *n* (missing) Yes (%)	4 (11) 1 (6.7)	2 (13) 2 (13.3)	–	–	3 (12) 2 (13.3)	1 (14) 1 (6.7)	–	3 (12) 1 (6.7)
36 months post initiation, *n* (missing) Yes (%)	3 (12) 0	5 (10) 2 (13.3)	4 (11) 4 (26.7)	3 (12) 3 (20.0)	1 (14) 0	–	1 (14) 1 (6.7)	3 (12) 2 (13.3)
48 months post initiation, *n* (missing) Yes (%)	4 (11) 1 (6.7)	1 (14) 1 (6.7)	1 (14) 1 (6.7)	1 (14) 1 (6.7)	–	–	2 (13) 2 (13.3)	3 (12) 2 (13.3)
60 months post initiation, *n* (missing) Yes (%)	1 (14) 0	2 (13) 2 (13.3)	–	–	1 (14) 1 (6.7)	1 (14) 1 (6.7)	–	1 (14) 1 (6.7)
72 months post initiation, *n* (missing) Yes (%)	1 (14) 0	–	–	1 (14) 1 (6.7)	–	–	–	–
84 months post initiation, *n* (missing) Yes (%)	1 (14) 0	2 (13) 1 (6.7)	–	1 (14) 1 (6.7)	1 (14) 1 (6.7)	1 (14) 1 (6.7)	1 (14) 1 (6.7)	3 (12) 1 (6.7)
96 months post initiation, *n* (missing) Yes (%)	1 (14) 0	2 (13) 1 (6.7)	–	–	–	1 (14) 1 (6.7)	–	1 (14) 1 (6.7)
108 months post initiation, *n* (missing) Yes (%)	–	1 (14) 1 (6.7)	–	1 (14) 1 (6.7)	–	–	–	–
120 months post initiation, *n* (missing) Yes (%)	–	2 (13) 2 (13.3)	–	1 (14) 1 (6.7)	–	–	–	–
132 months post initiation, *n* (missing) Yes (%)	–	1 (14) 0	–	–	–	–	–	–
144 months post initiation, *n* (missing) Yes (%)	–	–	–	–	–	–	–	–
156 months post initiation, *n* (missing) Yes (%)	1 (14) 1 (6.7)	–	–	–	–	–	1 (14) 1 (6.7)	–

Patients at each time point may differ from previous time points. Percentages are calculated with the denominator as the overall population for the cohort (*n* = 15).

There were no data available for the proportion of patients in younger sibling cohorts requiring surgery at index; however, 4 patients (26.7%) in Cohort 2, 3 patients (27.3%) in Cohort 3, and 5 patients (26.3%) in Cohort 5 presented with surgeries at index (Table 2 and Supplementary Tables S6 and S7). Where data were available, the number of patients requiring surgery after the initiation of idursulfase was similar between older and younger sibling cohorts (Table 2, Supplementary Fig. S4B, and Supplementary Tables S6 and S7).

At index, the proportion of patients with normal liver size determined by abdominal ultrasound was largely unknown for all cohorts (Table 2, Supplementary Fig. S4C and Supplementary Tables S6 and S7). Liver size determined by palpation was reported as ‘normal’ for 4 patients (26.7%) in Cohort 1 compared with 0 patients in Cohort 2 at index (Table 2, Supplementary Fig. S4D). Liver size determined by palpation was also more frequently reported as ‘normal’ at index in younger siblings than older siblings in the exploratory analyses (Cohort 1, *n* = 2 [18.2%] vs Cohort 3, *n* = 0; Supplementary Table S6, and Cohort 4, *n* = 6 [31.6%] vs Cohort 5, *n* = 0; Supplementary Table S7). The proportion of patients with normal liver size by palpation remained fairly consistent in all cohorts across the follow-up period (Table 2, Supplementary Fig. S4D, and Supplementary Tables S6 and S7).

Joint stiffness was less prevalent among younger siblings than older siblings at index, however, data for the rest of the follow-up period were limited (Supplementary Tables S8–S10). Data for the proportion of patients presenting with enlarged tonsils and adenoids and carpal tunnel syndrome after the initiation of idursulfase were sparse (Supplementary Tables S8–S10).

### 3.4 Treatment effect

GLMM analyses identified stronger effects (*p* < 0.05) for several outcomes post initiation of treatment with idursulfase for Cohort 1 versus Cohort 2 (Table 3). Cohort 1 had a greater height-for-age *Z*-score (coefficient 1.1; 95% CI: 0.4, 1.8; *p* = 0.002) and more measurements of normal liver size determined by palpation (coefficient 6.8; 95% CI: 2.3, 11.2; *p* = 0.004) than Cohort 2. Cohort 1 also had lower LVMI, a lower prevalence of cardiac valve disease, and fewer counts of enlarged tonsils and adenoids, joint stiffness, and carpal tunnel syndrome than Cohort 2. Similar results were observed in exploratory analyses, although a strong effect of treatment was not observed for joint stiffness in Cohort 1 versus Cohort 3 or cardiac valve disease in Cohort 4 versus Cohort 5 (*p* > 0.05; Supplementary Table S11).

**Table 3:** Generalized linear mixed model analyses for the primary objective (Cohort 1 vs Cohort 2; reference: older siblings).

Outcome measure	Coefficient (95% CI)	*p* value
Continuous variables (normal distribution; unity function)		
Urinary GAG levels,^a^ μg/mg	236.2 (−142.1, 614.5)	0.217
Height-for-age *Z*-score	1.1 (0.4, 1.8)	**0.002**
LVMI, g/m^2^	−43.7 (−73.2, −14.2)	**0.005**
Binary variables (binomial distribution; logit function)		
Cardiac valve disease	−1.7 (−3.1, −0.3)	**0.020**
Normal liver size (palpation)	6.8 (2.3, 11.2)	**0.004**
Normal liver size (ultrasound)	2.8 (−3.0, 8.5)	0.301
Count variables (Poisson distribution; log function)		
Enlarged tonsils and adenoids	−3.1 (−5.1, −1.1)	**0.003**
Joint stiffness	−2.3 (−4.2, −0.3)	**0.025**
Carpal tunnel syndrome	−3.8 (−6.4, −1.3)	**0.003**
Surgery	0.3 (−0.6, 1.2)	0.547

Strong effects showing differences between sibling cohorts (*p* < 0.05), which are characterized by a difference in treatment initiation and development of outcomes, are shown in bold font.

CI, confidence internal; GAG, glycosaminoglycan; GLMM, generalized linear mixed model; LVMI, left ventricular mass index.

a Higher urinary GAG levels were observed in the younger siblings when compared with the older siblings at treatment initiation, leading to higher mean urinary GAG levels in Cohort 1 vs Cohort 2 in the GLMM analysis.

Although point estimates suggested higher urinary GAG levels and more measurements of ‘normal liver size’ by ultrasound in younger versus older sibling cohorts (Table 3 and Supplementary Table S11), the effect of treatment was not strong (*p* > 0.05). Similarly, point estimates suggested that younger siblings in Cohort 1 had higher counts of surgeries than their older siblings in Cohorts 2 and 3, and that counts of surgeries were lower for younger siblings in Cohort 4 than for older siblings in Cohort 5. However, these observations were not significant (Table 3 and Supplementary Table S11).

## 4 Discussion

This retrospective study provides evidence of improved outcomes with early ERT (at ≤12 months of age) with idursulfase in sibling pairs with MPS II, who have at least a 12-month difference in age at the time of idursulfase initiation.

Younger siblings were consistently diagnosed earlier and treated earlier than their older siblings, possibly owing to increased awareness of MPS II by the family practice physician, which may have prompted family screening. Children with no family history of MPS II may experience substantial delays between disease onset and diagnosis [15]; indeed, the median age at diagnosis of MPS II in the Hunter Outcome Survey was roughly 3 years, while the median age at ERT initiation was 6.9 years [1]. Time to diagnosis is compounded by the fact that some symptoms in attenuated forms of MPS II may not manifest until several years after birth [3]. In these cases, presymptomatic MPS II can only be detected by newborn screening (NBS) [16]. Despite meeting the 1968 Wilson and Jungner criteria for screening [17], [18], NBS programs for MPS II are not yet universally available [19], [20]. Even when a diagnosis is made by NBS, decisions on treatment initiation may be challenging [21]. A recent Delphi consensus recommended that all patients, irrespective of their predicted phenotype, should initiate treatment as soon as possible following a confirmed diagnosis of MPS II [21]. The current study provides valuable evidence to support this recommendation, as well as supporting the expanded access of NBS programs for MPS II and highlighting the importance of broad disease awareness in settings where NBS is unavailable.

The overall findings demonstrated improved clinical outcomes with early ERT, with younger siblings trending towards having later occurrences of first events from birth for abnormal liver size, joint stiffness, enlarged tonsils and adenoids, and carpal tunnel syndrome. Lower frequency of musculoskeletal outcomes with early ERT (in patients aged younger than 16 months old) have also been reported in a recent international (non-US) retrospective chart review [22], supporting our findings.

For surgery, point estimates suggested a trend towards an earlier occurrence of first events in younger siblings than for older siblings in primary and both exploratory analyses. This finding should be interpreted with caution owing to the limited sample size, but it may suggest a lower threshold for referral and earlier scheduling or surgical interventions in younger siblings, owing to increased disease awareness following a familial diagnosis of MPS II. To test this hypothesis, future studies may analyze time-to-event measured from diagnosis rather than from birth.

Similarly, point estimates suggested an earlier occurrence of first events from birth of cardiac valve disease for younger siblings than for older siblings in the primary analysis and the exploratory analysis of Cohort 4 versus Cohort 5. However, these results were not statistically significant and should also be interpreted with caution given the small sample size. The observed trends may suggest ascertainment and management bias that leads to earlier diagnostic workup and more intensive surveillance in younger siblings following diagnosis of MPS II in an older sibling. This may include earlier and more frequent echocardiographies, which could increase the likelihood that valve abnormalities are identified and documented sooner. This is especially important because early cardiac disease in MPS II is often subtle [23], although its presence may indicate a more severe and rapidly progressing disease course [1], [24]. Accordingly, routine echocardiograms are recommended upon MPS II diagnosis [23]. Additionally, younger siblings may be assessed with more advanced technologies or practices, which could also have led to earlier documentation of first events. Results from the GLMM analysis showed that the overall prevalence of cardiac valve disease was lower among Cohort 1 than Cohort 2 and 3, suggesting that early treatment initiation may lead to improved outcomes. Long-term (≥ 5 years) treatment with ERT has been shown to significantly delay the progression of cardiac valve disease, but it is not possible to reverse established severe valvular involvement [25]. This highlights the importance of initiating ERT early in the disease course.

In a case study of disease progression in two brothers with MPS II, diagnosis of the older sibling prompted evaluation of the younger sibling; the older and younger siblings initiated treatment at 3 years 9 months and at 13 months, respectively. In this case, early treatment initiation in the younger sibling was associated with improved cardiac outcomes, a higher height-for-age *Z*-score, reduced joint stiffness, and greater neurocognitive benefits, although these findings are nuanced owing to differences in treatment history and clinical trial participation between the siblings [26]. Nevertheless, this broadly supports our own observations regarding the positive impact of early ERT on height-for-age *Z*-score, cardiac valve disease, and LVMI. Consistent with previous clinical trial and observational studies, we also observed stronger treatment effects in younger versus older siblings in liver size by palpation [2], [7], [9], [10]. Few studies have reported the effect of idursulfase on liver size by ultrasound, carpal tunnel syndrome, and enlarged tonsils and adenoids; this study contributes to the limited body of evidence for these outcomes in general, as well as in patients who initiate treatment before 12 months of age.

Urinary GAG levels are a useful biomarker of MPS II disease activity and response to ERT [[27], [28]]. Although point estimates suggested that younger siblings had higher urinary GAG levels than their older siblings at baseline, this was not significant. It is important to note that urinary excretion of GAGs is typically high in untreated young children and gradually decreases with age [[29], [30]]; therefore, baseline differences may primarily reflect the age at measurement rather than a true difference in disease activity. With treatment initiation, we found that observed urinary GAG levels decreased in both younger and older sibling cohorts, although we noted differences in the trajectories of urinary GAG decline between cohorts, such that younger siblings experienced a more rapid decline in urinary GAG levels. These findings should be interpreted with caution because they may reflect differences in established storage burden and tissue kinetics [[25], [31], [32]]. Because GAGs accumulate progressively [1], older patients may continue to mobilize and excrete stored GAGs for longer after treatment initiation, which may reflect a higher tissue GAG burden that delays the stabilization of urinary GAG levels. Conversely, when ERT is initiated earlier – before substantial GAG accumulation in tissues – urinary GAG levels may decline more rapidly and stabilize at lower levels. Considering this, the results do not, in isolation, demonstrate greater pharmacological efficacy when ERT is initiated earlier because we cannot disentangle treatment effect from age-related excretion patterns, baseline GAG storage burden, and timing of sampling. Similar observations were made in an international retrospective chart review study of patients who were diagnosed with MPS II before 72 months of age: younger patients (who initiated ERT at a mean [SD] age of 10.7 [4.6] months old; *n* = 11) had higher baseline urinary GAG levels than older patients (who initiated ERT at a mean [SD] age of 42.5 [15.3] months old; *n* = 81), yet a strong reduction in urinary GAG levels was observed in the overall cohort with initiation of ERT [22]. Although comparisons against age-specific reference values for urinary GAG concentrations may aid interpretation of these data, these are historically not universally available nor standardized across laboratories [33].

Real-world evidence has demonstrated that intravenous idursulfase is associated with longer survival for patients with MPS II [1], but studies comparing survival by age at treatment initiation are limited. Although the data presented here suggest that initiating ERT earlier may improve survival, this should be interpreted with caution owing to the longer follow-up times among older siblings. Although we did not actively collect safety data as part of the objectives, no adverse events were observed in the patients included in our study. The safety profile of idursulfase has been reported previously in the pivotal trial and case studies of ERT in patients younger than 18 months of age [[9], [11], [12], [34]].

An additional consideration is the slightly higher mean idursulfase dose administered in the younger sibling cohort. In clinical practice, particularly in very young children with low body weight, clinicians may use full 6 mg vials to avoid medication wastage, which can result in doses exceeding 0.5 mg/kg; no adverse events related to ERT have been observed with higher doses [13]. In our study, this off-label practice occurred at a single site where three younger siblings received an initial dose of 1.2 mg/kg weekly, contributing to a higher mean dose in the younger than in older sibling cohorts. However, given the small number of patients affected and the modest difference in mean dose between cohorts, it is unlikely that dosing differences meaningfully influenced outcomes. This is largely consistent with evidence from the Phase 1/2 trial of idursulfase, which demonstrated no clinical meaningful differences in safety, other than infusion-related reactions, across a range of idursulfase doses administered every other week [35].

ERT is a well-established therapeutic strategy for MPS II, and accumulating evidence from this study and others suggests that early initiation may improve clinical outcomes [11], [12], [13]. For patients who are diagnosed prenatally, in utero ERT offers the earliest chance of treatment and is currently being investigated in the Phase 1 PEARL study (NCT04532047) [[36], [37]]. However, until this delivery method is approved, early administration of ERT remains the best approach.

### 4.1 Strengths and limitations

This study was designed to cover a wide geography so that findings may be generalizable to populations with MPS II in a variety of regions. To reduce the impact of potential differences in classification, data were collected from raw clinical variables rather than classification outcomes, enabling more robust comparisons between sibling pairs. Utilizing a controlled study design such as matching (which is difficult for rare diseases), was an important approach to addressing the study objective to control confounding bias. Because siblings were similar in terms of genetic disposition and sociodemographic variables, potential unobservable (clinical and sociodemographic) variables could be better controlled to minimize confounding bias. However, a case series has demonstrated intrafamilial variability in the presentation and clinical course of MPS II between patients with the same *IDS* variant [38], which may limit complete control of confounding bias between siblings.

Although this is one of the largest sibling set analyses to date investigating the impact of early treatment initiation in MPS II, there are some important limitations. Owing to the retrospective study design, clinical data were missing for some of the siblings; there was also a lack of data for cognitive impairment. Two sites in France and the UK required informed consent forms, which were waived in other countries; this may have introduced selection bias at these sites. Different lengths of follow-up time between siblings limited the ability to compare the number of events and severity of disease between siblings at equivalent ages. It should be noted that time-to-event analyses may be influenced by the shorter follow-up times in the younger sibling cohorts because they were diagnosed and treated earlier than their older siblings. Although cumulative first events from age at diagnosis and age at treatment initiation were assessed for a few variables, insufficient overlaps in follow-up times between younger and older sibling cohorts prevented meaningful analysis. Furthermore, the effect of left censoring cannot be excluded, because information for older siblings between birth and treatment initiation was more likely to be missing compared with younger siblings. It is therefore uncertain whether the number of events would have converged between younger and older siblings with equivalent follow-up and data availability; additional studies with longer follow-up periods are warranted to confirm this. Trends for clinical outcomes pertaining to time-to-event from first diagnosis may have been missed, because only Kaplan–Meier curves for time from birth were produced in these time-to-event analyses. To avoid issues of multicollinearity, only two variables (sibling status and age at time of measurement) were entered in the GLMM model; the model does not account for differing durations of treatment between siblings. The modest sample size and volume of missing data contributed to wide confidence intervals and a lack of statistical significance for several comparisons in the time-to-event and GLMM analyses, thereby limiting the power to detect meaningful differences between siblings. However, the overall findings do not contradict the known efficacy profile of idursulfase.

## 5 Conclusions

In conclusion, this study provides evidence that early diagnosis of MPS II may facilitate early treatment with idursulfase. Early treatment may prevent and reduce the risk of developing certain outcomes including joint stiffness, increased LVMI, cardiac valve disease, carpal tunnel syndrome, enlarged tonsils and adenoids, and abnormal liver size, and may increase height-for-age *Z*-score. While the underlying age-related differences in urinary GAG concentrations at baseline complicated the interpretation of GLMM analyses, the absolute levels of urinary GAGs clearly dropped following ERT initiation in all cohorts, with the largest reductions observed in the cohorts where treatment was initiated at younger ages. Despite the small sample size and limited data availability, the findings are in line with the known efficacy profile of idursulfase. This study contributes to the limited but growing body of evidence for the real-world effectiveness of early initiation of idursulfase treatment in patients with MPS II.

## CRediT authorship contribution statement

**Barbara K. Burton:** Writing – review & editing, Methodology, Investigation, Conceptualization. **Bernd Schweikert:** Writing – review & editing, Methodology, Formal analysis, Data curation. **Hernàn Amartino:** Writing – review & editing, Investigation. **Maira Sharipova:** Writing – review & editing, Investigation. **Jaime Lopez-Valdez:** Writing – review & editing, Investigation. **Deniz Kor:** Writing – review & editing, Investigation. **Huseyin Bilgin:** Writing – review & editing, Investigation. **Walla Al-Hertani:** Writing – review & editing, Investigation. **Ana Maria Martins:** Writing – review & editing, Investigation. **Dimitrios Zafeiriou:** Writing – review & editing, Investigation. **Nathalie Guffon:** Writing – review & editing, Investigation. **Olivia Okoli:** Writing – review & editing, Formal analysis, Data curation. **Diana Sanchez-Hernandez:** Writing – review & editing, Formal analysis, Data curation. **Jennifer Audi:** Writing – review & editing, Conceptualization. **Jaco Botha:** Writing – review & editing, Methodology, Conceptualization. **Csaba Siffel:** Writing – review & editing, Validation, Supervision, Methodology, Formal analysis, Conceptualization. **Olulade Ayodele:** Writing – review & editing, Validation, Supervision, Methodology, Conceptualization.

## Ethics approval and consent to participate

This study was conducted in accordance with ethical principles that have their origin in the Declaration of Helsinki, Good Clinical Practice (GCP), the International Society for Pharmacoepidemiology (ISPE) Guidelines for Good Pharmacoepidemiology Practice (GPP), and any applicable local regulations. The final protocol, any amendments, and informed consent documentation were submitted, reviewed, and approved by an Institutional Review Board (IRB) and/or Institutional Ethics Committee (IEC) for each site participating in the study.

## Ethics declaration

### Informed consent and patient details

Written informed consent to take part in the study and to publish the article has been obtained from all participants or their legal representatives. The privacy rights of participants have been observed.

### Studies in human

This study was performed in compliance with relevant laws, regulatory frameworks and guidelines where the research took place.

The Local IRB/ethical committee in each participating country granted an exemption. The authors provided the following explanation: As this was a non-interventional study that only abstracted non-personal identifiable data retrospectively from existing medical charts, IRB(s)/EC(s) in all countries were requested to approve a waiver for the informed consent. Where the request for a waiver of consent was rejected, then the investigator gave the patient (and, if applicable, parent or legal guardian) oral and written information about the study in a form that the patient (and, if applicable, parent or legal guardian) can understand, and obtained the patient's written consent (or, if applicable, the patient's assent and the parent's or legal guardian's written consent) before collection of patient data.

## Funding

This study was funded by Takeda Pharmaceutical Company Limited.

## Declaration of competing interest

B.K.B has received consulting fees and/or participated in advisory boards for Agios Pharmaceuticals, Alexion, Alltrna, Amgen, Applied Therapeutics, Aro Biotherapeutics, BioMarin Pharmaceutical, Chiesi Farmaceutici, JCR Pharmaceuticals, Jnana Therapeutics, Moderna, Orchard Therapeutics, Passage Bio, Sanofi Genzyme, Takeda, Travere, and Ultragenyx. She has performed contracted research for Takeda and has been involved in company-sponsored clinical trials with BioMarin Pharmaceutical, Denali Therapeutics, Homology Medicines, JCR Pharmaceuticals, Jnana Therapeutics, Sangamo Therapeutics, Synlogic, Takeda, and Ultragenyx. B.S., O.O., and D.S-H. are full-time employees of ICON plc., the clinical research organization commissioned by Takeda to perform this study. H.A. has received consulting fees, fees for service, and/or participated in advisory boards for Amicus Therapeutics, BioMarin Pharmaceutical, Janssen Pharmaceuticals, PTC Therapeutics, Sanofi Genzyme, Takeda, and Ultragenyx. He has performed contracted research for Allievex, Amicus Therapeutics, bluebird bio, JCR Pharmaceuticals, Minoryx Therapeutics, PTC Therapeutics, Sanofi Genzyme, and Takeda. A.M.M. has received advisory board honoraria from Astra Zeneca-Alexion Pharmaceuticals, BioMarin Pharmaceutical, JCR Pharmaceuticals, and Sanofi. She has also received honoraria and travel support from Astra Zeneca-Alexion Pharmaceuticals, BioMarin Pharmaceutical, Chiesi Pharmaceuticals, JCR Pharmaceuticals, and Sanofi. D.Z. has received consulting fees, travel and lecture honoraria, and participated in advisory boards for BioMarin Pharmaceutical, Chiesi, PTC Therapeutics, Sanofi Genzyme, and Takeda. N.G. has received consulting payments and travel support and has participated as a clinical trial investigator in MARS for BioMarin Pharmaceutical Inc.; is a Principal Investigator in clinical trials for Chiesi, JCR Pharmaceuticals, Sanofi, Takeda Pharmaceuticals, and Ultragenyx; and has received consulting/speaker fees from Chiesi, JCR Pharmaceuticals, Sanofi, and Ultragenyx. J.A. was an employee of and held stock/stock options of Takeda Pharmaceutical Company Limited at the time of this analysis. J.B., C.S., and O.A. are full-time employees and hold stock/stock options of Takeda Pharmaceuticals Company Limited. M.S., J.L-V., D.K., H.B., and W.A-H. declare no relevant conflicts of interest that relate to the research described in this paper. If there are other authors, they declare that they have no known competing financial interests or personal relationships that could have appeared to influence the work reported in this paper.

## Data Availability

The datasets, including the redacted study protocol, redacted statistical analysis plan, and individual participants' data supporting the results reported in this article, will be made available to researchers who provide a methodologically sound proposal. The data will be provided after their de-identification, in compliance with applicable privacy laws, data protection, and requirements for consent and anonymization.
