# Supplementary material for: Effectiveness of early initiation of idursulfase in infants and very young children with mucopolysaccharidosis II: a retrospective chart review in siblings
Source: Mol Genet Metab Rep. 2026 Sep 25;49:101354. doi: 10.1016/j.ymgmr.2026.101354 (PMC13635862; doi:10.1016/j.ymgmr.2026.101354)
Supplement: Supplementary file 1 [file mmc1.docx]

# Supplementary materials

### Supplementary Table S1. Study variables and outcomes

**Exposures**

- Patient’s age at treatment initiation
- Dosing and frequency of treatment
- Adherence to treatment^a^

**Clinical Outcomes**

- Signs and symptoms by organ system
- Presence of symptoms
- Age at onset
- Frequency of recurrence
- Severity
- Urinary GAG levels
- Growth parameters
- Height and corresponding Z-scores
- Weight and corresponding Z-scores
- Cardiac parameters
- LVMI^b^
- Onset, type, severity, and progression of cardiac valve disease^b^
- Respiratory parameters
- Hospitalizations for respiratory infections
- Frequency and severity of respiratory infections and associated antibiotic use
- Ear, nose, and throat parameters
- Frequency and severity of ear infections and associated treatment utilization (including antibiotic use)
- Presence and age at onset of enlarged tonsils and adenoids and associated treatment and outcomes (including reoccurrence)
- Presence and age at onset of obstructive sleep apnea
- Presence and severity of hearing problems and associated treatment utilization (including T-tubes insertion and hearing aids) and outcomes
- Abdominal and gastrointestinal parameters
- Liver and spleen size^c^
- Presence of chronic diarrhea
- Musculoskeletal parameters
- Joint stiffness^d^
- Presence and age at onset of claw hands
- Presence and age at onset of coarse facial features
- Presence and description of dysostosis multiplex (including radiology findings)
- Presence and age at onset of spinal abnormality (scoliosis, kyphosis)
- Presence and age at onset of immobility
- Incidence and severity of vertebral collapse (including associated nerve damage)
- Neurological parameters
- Presence and age at onset of carpal tunnel syndrome, its treatment and outcomes (including reoccurrence)
- Presence and age at onset of peripheral nerve (non-carpal tunnel) involvement, including sensorimotor investigations (e.g. evoked potentials)
- Presence and age at onset of neurocognitive decline
- Diagnosis of cognitive impairment (including age at diagnosis, method of diagnosis, and results of formal testing, if applicable)
- Other parameters
- Frequency and causes of hospitalizations
- Frequency and causes of admissions to the emergency room
- Frequency and reasons for surgeries (including types and specific surgeries performed)

**Other study variables**

- Age at MPS II diagnosis
- Enzyme level at treatment baseline
- *IDS* genetic mutation
- Idursulfase discontinuation (including age at discontinuation and reasons for discontinuation, if applicable)
- HSCT treatment (if applicable)
- Death (including age at time of death and cause of death, if applicable)

^a^Defined as receiving > 80% of scheduled infusions. ^b^As calculated by echocardiography. ^c^As estimated by palpation and/or abdominal ultrasound. ^d^In wrists, elbows, shoulders, hips, knees, and/or ankles.
GAG, glycosaminoglycan; HSCT, hematopoietic stem cell transplantation; *IDS*, *iduronate-2-sulfatase*; LVMI, left ventricular mass index; MPS II, mucopolysaccharidosis II.

### Supplementary Table S2. Patient characteristics (exploratory analysis cohorts)

|  | **Younger sibling subgroup** | **Older sibling subgroups** | |
| --- | --- | --- | --- |
| **Characteristic** | **Cohort 4 *n* = 19** | **Cohort 3  *n* = 11** | **Cohort 5**  ***n* = 19** |
| Median (P10–P90) age at MPS II diagnosis, months | 3 (0–14) | 44 (35–81) | 36 (21–81) |
| Median (P10–P90) age at idursulfase initiation, months | 6.0 (1–19) | 60 (39–84) | 44 (28–84) |
| Median (P10–P90) follow-up duration from index, months | 49 (34–151) | 96 (78–143) | 93 (39–146) |
| Cognitive impairment at time of data collection, *n* (%)  Diagnosed by clinical judgment, *n* (%)  Diagnosed by formal testing, *n* (%) | 9 (47.4)  6 (31.6)  3 (15.8) | 10 (90.9)  6 (54.5)  4 (36.4) | 16 (84.2)  11 (57.9)  5 (26.3) |
| Deceased, *n* (%)  Median (P10–P90) age at death, months | 2 (10.5)  104.5 (43–166) | 5 (45.5)  169 (120–205) | 6 (31.6)  150.5 (120–205) |
| Cause of death, *n* (%)  Acute respiratory insufficiency  Central apnea  Mechanical complication of tracheostomy  MPS II disease evolution  Pneumonia | 0  0  0  1 (5.3)  1 (5.3) | 1 (9.1)  0  1 (9.1)  1 (9.1)  2 (18.2) | 1 (5.3)  1 (5.3)  1 (5.3)  1 (5.3)  2 (10.5) |
| Mean (SD) idursulfase dose at initiation, mg/kg | 0.6 (0.3) | 0.5 (0.0) | 0.5 (0.1) |
| Adherence to treatment,^a^ *n* (%) | 17 (89.5) | 9 (81.8) | 17 (89.5) |
| Discontinued treatment, *n* (%)  Median (P10–P90) age at discontinuation, months | 2 (10.5)  31 (26–36) | 1 (9.1)  181 (–) | 4 (21.1)  114 (52–181) |
| Reason for discontinuation, *n* (%)  Enrolled in clinical trial  Death  Neurological disease | 2 (10.5)  0  0 | 0  1 (9.1)  0 | 2 (10.5)  1 (5.3)  1 (5.3) |
| Continued treatment, *n* (%) | 16 (84.2) | 10 (90.9) | 14 (73.7) |
| Treatment continuation unknown, *n* (%) | 1 (5.3) | 0 | 1 (5.3) |
| Pathogenic *IDS* genetic variant, *n* (%)  Missense  Splicing^b^  Nonsense  Frameshift  Deletion  Recombination  Not reported | 9 (47.4)  5 (26.3)  2 (10.5)  1 (5.3)  1 (5.3)  1 (5.3)  0 | 6 (54.5)  3 (27.3)  0  1 (9.1)  0  0  1 (9.1) | 9 (47.4)  4 (21.1)  2 (10.5)  1 (5.3)  1 (5.3)  1 (5.3)  1 (5.3) |

^a^Defined as receiving > 80% of infusions. ^b^One pair of siblings had a missense mutation (single nucleotide substitution) that disrupted splicing; they were included in the splicing category.
*IDS*, *iduronate-2-sulfatase*; MPS II, mucopolysaccharidosis II; P10, 10th percentile; P90, 90th percentile; SD, standard deviation.

### Supplementary Table S3. Clinical outcomes for continuous variables (primary analysis: Cohort 1 vs Cohort 2)

|  | **Urinary GAG levels, μg/mg** | | **Height-for-age Z-score** | | **LVMI, g/m^2^** | |
| --- | --- | --- | --- | --- | --- | --- |
| **Time of outcome measurement** | **Cohort 1**  ***n* = 15** | **Cohort 2**  ***n* = 15** | **Cohort 1**  ***n* = 15** | **Cohort 2**  ***n* = 15** | **Cohort 1**  ***n* = 15** | **Cohort 2**  ***n* = 15** |
| Index, *n* (missing)  Mean (SD) | 7 (8)  1121.0 (754.3) | 7 (8)  380.3 (199.6) | 11 (4)  0.6 (1.4) | 10 (5)  −0.7 (2.1) | 2 (13)  89.4 (36.8) | 5 (10)  97.4 (35.5) |
| 12 months post initiation, *n* (missing)  Mean (SD) | 4 (11)  152.8 (113.9) | 1 (14)  41.5 (–) | 4 (11)  1.0 (1.3) | 3 (12)  −0.4 (1.8) | 1 (14)  62.4 (–) | 2 (13)  214.2 (152.3) |
| 24 months post initiation, *n* (missing)  Mean (SD) | 2 (13)  43.8 (13.2) | 4 (11)  166.3 (101.3) | 5 (10)  0.3 (2.3) | 6 (9)  −0.1 (1.6) | 2 (13)  56.3 (3.7) | – |
| 36 months post initiation, *n* (missing)  Mean (SD) | 3 (12)  123.8 (145.5) | 3 (12)  65.1 (38.6) | 3 (12)  −0.1 (1.7) | 4 (11)  0.9 (1.6) | – | 1 (14)  72.4 (–) |
| 48 months post initiation, *n* (missing)  Mean (SD) | 1 (14)  21.1 (–) | 2 (13)  119.6 (131.2) | 4 (11)  0.7 (0.8) | 3 (12)  0.4 (2.3) | 1 (14)  83.2 (–) | – |
| 60 months post initiation, *n* (missing)  Mean (SD) | 2 (13)  73.0 (74.4) | 2 (13)  79.0 (49.8) | 2 (13)  0.1 (1.2) | 4 (11)  −0.7 (2.2) | 1 (14)  77.0 (–) | – |
| 72 months post initiation, *n* (missing)  Mean (SD) | 2 (13)  61.9 (65.2) | 4 (11)  69.2 (46.9) | 1 (14)  1.0 (–) | 3 (12)  −1.1 (1.9) | 1 (14)  60.0 (–) | – |
| 84 months post initiation, *n* (missing)  Mean (SD) | 2 (13)  58.9 (55.6) | 3 (12)  214.7 (262.6) | 1 (14)  −3.2 (–) | 4 (11)  −1.4 (2.4) | 1 (14)  76.3 (–) | 1 (14)  70.0 (–) |
| 96 months post initiation, *n* (missing)  Mean (SD) | – | 1 (14)  123.0 (–) | – | 2 (13)  −0.7 (2.6) | – | 2 (13)  54.0 (35.4) |
| 108 months post initiation, *n* (missing)  Mean (SD) | 2 (13)  84.1 (28.8) | – | 1 (14)  −0.5 (–) | 1 (14)  −2.6 (–) | – | 1 (14)  80.0 (–) |
| 120 months post initiation, *n* (missing)  Mean (SD) | 1 (14)  51.3 (–) | – | – | 2 (13)  −2.2 (1.2) | – | 2 (13)  99.6 (20.6) |
| 132 months post initiation, *n* (missing)  Mean (SD) | – | – | – | 1 (14)  −3.2 (–) | – | 1 (14)  72.0 (–) |
| 144 months post initiation, *n* (missing)  Mean (SD) | 1 (14)  46.9 (–) | – | 1 (14)  −1.7 (–) | 1 (14)  −3.2 (–) | – | – |
| 156 months post initiation, *n* (missing)  Mean (SD) | 1 (14)  33.6 (–) | – | – | – | 1 (14)  92.5 (–) | – |

Patients at each time point may differ from previous time points.
SD, standard deviation.

### Supplementary Table S4. Clinical outcomes for continuous variables (exploratory analysis: Cohort 1 vs Cohort 3)

|  | **Urinary GAG levels, μg/mg** | | **Height-for-age Z-score** | | **LVMI, g/m^2^** | |
| --- | --- | --- | --- | --- | --- | --- |
| **Time of outcome measurement** | **Cohort 1**  ***n* = 11** | **Cohort 3**  ***n* = 11** | **Cohort 1**  ***n* = 11** | **Cohort 3**  ***n* = 11** | **Cohort 1**  ***n* = 11** | **Cohort 3**  ***n* = 11** |
| Index, *n* (missing)  Mean (SD) | 4 (7)  987.6 (995.5) | 4 (7)  311.3 (226.9) | 7 (4)  0.0 (1.4) | 6 (5)  −1.4 (1.9) | 1 (10)  115.4 (–) | 3 (8)  115.3 (33.5) |
| 12 months post initiation, *n* (missing)  Mean (SD) | 4 (7)  152.8 (113.9) | 1 (10)  41.5 (–) | 4 (7)  1.0 (1.3) | 3 (8)  −0.4 (1.8) | 1 (10)  62.4 (–) | 2 (9)  214.2 (152.3) |
| 24 months post initiation, *n* (missing)  Mean (SD) | 2 (9)  43.8 (13.2) | 4 (7)  166.3 (101.3) | 3 (8)  −0.3 (3.0) | 5 (6)  −0.5 (1.5) | 2 (9)  56.3 (3.7) | – |
| 36 months post initiation, *n* (missing)  Mean (SD) | 2 (9)  154.7 (191.3) | 3 (8)  65.1 (38.6) | 2 (9)  −0.7 (1.9) | 3 (8)  0.6 (1.7) | – | 1 (10)  72.4 (–) |
| 48 months post initiation, *n* (missing)  Mean (SD) | 1 (10)  21.1 (–) | 2 (9)  119.6 (131.2) | 2 (9)  0.5 (0.9) | 3 (8)  0.4 (2.3) | – | – |
| 60 months post initiation, *n* (missing)  Mean (SD) | 2 (9)  73.0 (74.4) | 2 (9)  79.0 (49.8) | 2 (9)  0.1 (1.2) | 3 (8)  −1.3 (2.1) | 1 (10)  77.0 (–) | – |
| 72 months post initiation, *n* (missing)  Mean (SD) | 2 (9)  61.9 (65.2) | 3 (8)  54.2 (44.2) | 1 (10)  1.0 (–) | 2 (9)  −0.2 (1.7) | 1 (10)  60.0 (–) | – |
| 84 months post initiation, *n* (missing)  Mean (SD) | 2 (9)  58.9 (55.6) | 3 (8)  214.7 (262.6) | 1 (10)  −3.2 (–) | 3 (8)  −2.1 (2.4) | 1 (10)  76.3 (–) | 1 (10)  70.0 (–) |
| 96 months post initiation, *n* (missing)  Mean (SD) | – | 1 (10)  123.0 (–) | – | 1 (10)  −2.5 (–) | – | 2 (9)  54.0 (35.4) |
| 108 months post initiation, *n* (missing)  Mean (SD) | 2 (9)  84.1 (28.8) | – | 1 (10)  −0.5 (–) | 1 (10)  −2.6 (–) | – | 1 (10)  80.0 (–) |
| 120 months post initiation, *n* (missing)  Mean (SD) | 1 (10)  51.3 (–) | – | – | 2 (9)  −2.2 (1.2) | – | 2 (9)  99.6 (20.6) |
| 132 months post initiation, *n* (missing)  Mean (SD) | – | – | – | 1 (10)  −3.2 (–) | – | 1 (10)  72.0 (–) |
| 144 months post initiation, *n* (missing)  Mean (SD) | 1 (10)  46.9 (–) | – | 1 (10)  −1.7 (–) | 1 (10)  −3.2 (–) | – | – |
| 156 months post initiation, *n* (missing)  Mean (SD) | 1 (10)  33.6 (–) | – | – | – | 1 (10)  92.5 (–) | – |

Patients at each time point may differ from previous time points.
SD, standard deviation.

### Supplementary Table S5. Clinical outcomes for continuous variables (exploratory analysis: Cohort 4 vs Cohort 5)

|  | **Urinary GAG levels, μg/mg** | | **Height-for-age Z-score** | | **LVMI, g/m^2^** | |
| --- | --- | --- | --- | --- | --- | --- |
| **Time of outcome measurement** | **Cohort 4**  ***n* = 19** | **Cohort 5**  ***n* = 19** | **Cohort 4**  ***n* = 19** | **Cohort 5**  ***n* = 19** | **Cohort 4**  ***n* = 19** | **Cohort 5**  ***n* = 19** |
| Index, *n* (missing)  Mean (SD) | 9 (10)  1053.7 (692.8) | 9 (10)  468.6 (251.0) | 15 (4)  0.7 (1.5) | 14 (5)  −0.7 (2.1) | 4 (15)  73.4 (31.5) | 6 (13)  85.8 (42.6) |
| 12 months post initiation, *n* (missing)  Mean (SD) | 4 (15)  152.8 (113.9) | 2 (17)  160.1 (167.8) | 5 (14)  0.3 (1.9) | 5 (14)  −0.5 (1.4) | 1 (18)  62.4 (–) | 2 (17)  214.2 (152.3) |
| 24 months post initiation, *n* (missing)  Mean (SD) | 2 (17)  43.8 (13.2) | 5 (14)  176.5 (90.7) | 5 (14)  0.3 (2.3) | 7 (12)  −0.2 (1.5) | 2 (17)  56.3 (3.7) | – |
| 36 months post initiation, *n* (missing)  Mean (SD) | 4 (15)  136.4 (121.5) | 4 (15)  91.8 (61.9) | 5 (14)  0.3 (1.6) | 6 (13)  0.3 (1.5) | – | 1 (18)  72.4 (–) |
| 48 months post initiation, *n* (missing)  Mean (SD) | 1 (18)  21.1 (–) | 2 (17)  119.6 (131.2) | 5 (14)  0.4 (0.9) | 4 (15)  0.1 (2.0) | 1 (18)  83.2 (–) | – |
| 60 months post initiation, *n* (missing)  Mean (SD) | 2 (17)  73.0 (74.4) | 2 (17)  79.0 (49.8) | 2 (17)  0.1 (1.2) | 4 (15)  −0.7 (2.2) | 1 (18)  77.0 (–) | – |
| 72 months post initiation, *n* (missing)  Mean (SD) | 2 (17)  61.9 (65.2) | 4 (15)  59.2 (46.9) | 1 (18)  1.0 (–) | 3 (16)  −1.1 (1.9) | 1 (18)  60.0 (–) | – |
| 84 months post initiation, *n* (missing)  Mean (SD) | 2 (17)  58.9 (55.6) | 3 (16)  214.7 (262.6) | 2 (17)  −2.8 (0.6) | 4 (15)  −1.4 (2.4) | 1 (18)  76.3 (–) | 1 (18)  70.0 (–) |
| 96 months post initiation, *n* (missing)  Mean (SD) | – | 1 (18)  123.0 (–) | – | 2 (17)  −0.7 (2.6) | – | 2 (17)  54.0 (35.4) |
| 108 months post initiation, *n* (missing)  Mean (SD) | 2 (17)  84.1 (28.8) | – | 1 (18)  −0.5 (–) | 1 (18)  −2.6 (–) | – | 1 (18)  80.0 (–) |
| 120 months post initiation, *n* (missing)  Mean (SD) | 1 (18)  51.3 (–) | – | – | 2 (17)  −2.2 (1.2) | – | 2 (17)  99.6 (20.6) |
| 132 months post initiation, *n* (missing)  Mean (SD) | – | – | – | 1 (18)  −3.2 (–) | – | 1 (18)  72.0 (–) |
| 144 months post initiation, *n* (missing)  Mean (SD) | 1 (18)  46.9 (–) | – | 1 (18)  −1.7 (–) | 1 (18)  −3.2 (–) | – | – |
| 156 months post initiation, *n* (missing)  Mean (SD) | 1 (18)  33.6 (–) | – | – | – | 1 (18)  92.5 (–) | – |

Patients at each time point may differ from previous time points.
SD, standard deviation.

### Supplementary Table S6. Clinical outcomes for cardiac valve disease, surgery, and normal liver size (exploratory analysis: Cohort 1 vs Cohort 3)

|  | **Cardiac valve disease** | | **Surgery** | | **Normal liver size (ultrasound)** | | **Normal liver size (palpation)** | |
| --- | --- | --- | --- | --- | --- | --- | --- | --- |
| **Time of outcome measurement** | **Cohort 1**  ***n* = 11** | **Cohort 3**  ***n* = 11** | **Cohort 1**  ***n* = 11** | **Cohort 3**  ***n* = 11** | **Cohort 1**  ***n* = 11** | **Cohort 3**  ***n* = 11** | **Cohort 1**  ***n* = 11** | **Cohort 3**  ***n* = 11** |
| Index, *n* (missing)  Yes (%) | 5 (6)  2 (18.2) | 8 (3)  4 (36.4) | – | 3 (8)  3 (27.3) | 2 (9)  1 (9.1) | 4 (7)  2 (18.2) | 3 (8)  2 (18.2) | 5 (6)  0 |
| 12 months post initiation, *n* (missing)  Yes (%) | 2 (9)  0 | 2 (9)  2 (18.2%) | 1 (10)  1 (9.1) | 1 (10)  1 (9.1) | 2 (9)  1 (9.1) | – | 2 (9)  1 (9.1) | 2 (9)  0 |
| 24 months post initiation, *n* (missing)  Yes (%) | 4 (7)  1 (9.1) | – | – | – | 2 (9)  1 (9.1) | – | – | 3 (8)  1 (9.1) |
| 36 months post initiation, *n* (missing)  Yes (%) | 2 (9)  0 | 4 (7)  2 (18.2) | 3 (8)  3 (27.3) | 2 (9)  2 (18.2) | 1 (10)  0 | – | – | 2 (9)  1 (9.1) |
| 48 months post initiation, *n* (missing)  Yes (%) | 2 (9)  1 (9.1) | 1 (1)  1 (9.1) | 1 (10)  1 (9.1) | 1 (10)  1 (9.1) | – | – | – | 3 (8)  2 (18.2) |
| 60 months post initiation, *n* (missing)  Yes (%) | 1 (10)  0 | 2 (9)  2 (18.2) | – | – | 1 (10)  1 (9.1) | 1 (10)  1 (9.1) | – | – |
| 72 months post initiation, *n* (missing)  Yes (%) | 1 (10)  0 | – | – | – | – | – | – | – |
| 84 months post initiation, *n* (missing)  Yes (%) | 1 (10)  0 | 1 (10)  1 (9.1) | – | 1 (10)  1 (9.1) | 1 (10)  1 (9.1) | 1 (10)  1 (9.1) | 1 (10)  1 (9.1) | 1 (10)  0 |
| 96 months post initiation, *n* (missing)  Yes (%) | 1 (10)  0 | 2 (9)  1 (9.1) | – | – | – | 1 (10)  1 (9.1) | – | – |
| 108 months post initiation, *n* (missing)  Yes (%) | – | 1 (10)  1 (9.1) | – | 1 (10)  1 (9.1) | – | – | – | – |
| 120 months post initiation, *n* (missing)  Yes (%) | – | 2 (9)  2 (18.2) | – | 1 (10)  1 (9.1) | – | – | – | – |
| 132 months post initiation, *n* (missing)  Yes (%) | – | 1 (10)  0 | – | – | – | – | – | – |
| 144 months post initiation, *n* (missing)  Yes (%) | – | – | – | – | – | – | – | – |
| 156 months post initiation, *n* (missing)  Yes (%) | 1 (10)  1 (9.1) | – | – | – | – | – | 1 (10)  1 (9.1) | – |

Patients at each time point may differ from previous time points. Percentages are calculated with the denominator as the overall population for the cohort (*n* = 11).

### Supplementary Table S7. Clinical outcomes for cardiac valve disease, surgery and normal liver size (exploratory analysis: Cohort 4 vs Cohort 5)

|  | **Cardiac valve disease** | | **Surgery** | | **Normal liver size (ultrasound)** | | **Normal liver size (palpitation)** | |
| --- | --- | --- | --- | --- | --- | --- | --- | --- |
| **Time of outcome measurement** | **Cohort 4**  ***n* = 19** | **Cohort 5**  ***n* = 19** | **Cohort 4**  ***n* = 19** | **Cohort 5**  ***n* = 19** | **Cohort 4**  ***n* = 19** | **Cohort 5**  ***n* = 19** | **Cohort 4**  ***n* = 19** | **Cohort 5**  ***n* = 19** |
| Index, *n* (missing)  Yes (%) | 10 (9)  6 (31.6) | 12 (7)  7 (36.8) | – | 5 (14)  5 (26.3) | 2 (17)  1 (5.3) | 7 (12)  2 (10.5) | 10 (9)  6 (31.6) | 9 (10)  0 |
| 12 months post initiation, *n* (missing)  Yes (%) | 5 (14)  0 | 2 (17)  2 (10.5) | 1 (18)  1 (5.3) | 2 (17)  2 (10.5) | 2 (17)  1 (5.3) | 1 (18)  1 (5.3) | 2 (17)  1 (5.3) | 3 (16)  1 (5.3) |
| 24 months post initiation, *n* (missing)  Yes (%) | 4 (15)  1 (5.3) | 2 (17)  2 (10.5) | 1 (18)  1 (5.3) | – | 3 (16)  2 (10.5) | 1 (18)  1 (5.3) | – | 4 (15)  2 (10.5) |
| 36 months post initiation, *n* (missing)  Yes (%) | 3 (16)  0 | 5 (14)  2 (10.5) | 4 (15)  4 (21.1) | 3 (16)  3 (15.8) | 1 (18)  0 | – | 1 (18)  1 (5.3) | 4 (15)  2 (10.5) |
| 48 months post initiation, *n* (missing)  Yes (%) | 4 (15)  1 (5.3) | 1 (18)  1 (5.3) | 1 (18)  1 (5.3) | 1 (18)  1 (5.3) | – | – | 3 (16)  3 (15.8) | 3 (16)  2 (10.5) |
| 60 months post initiation, *n* (missing)  Yes (%) | 1 (18)  0 | 2 (17)  2 (10.5) | – | – | 1 (18)  1 (5.3) | 1 (18)  1 (5.3) | – | 1 (18)  1 (5.3) |
| 72 months post initiation, *n* (missing)  Yes (%) | 1 (18)  0 | – | – | 1 (18)  1 (5.3) | – | – | – | – |
| 84 months post initiation, *n* (missing)  Yes (%) | 1 (18)  0 | 2 (17)  1 (5.3) | – | 1 (18)  1 (5.3) | 1 (18)  1 (5.3) | 1 (18)  1 (5.3) | 1 (18)  1 (5.3) | 3 (16)  1 (5.3) |
| 96 months post initiation, n (missing)  Yes (%) | 1 (18)  0 | 3 (16)  2 (10.5) | – | – | – | 1 (18)  1 (5.3) | – | 1 (18)  1 (5.3) |
| 108 months post initiation, *n* (missing)  Yes (%) | – | 1 (18)  1 (5.3) | – | 1 (18)  1 (5.3) | – | – | – | – |
| 120 months post initiation, *n* (missing)  Yes (%) | – | 2 (17)  2 (10.5) | – | 1 (18)  1 (5.3) | – | – | – | – |
| 132 months post initiation, *n* (missing)  Yes (%) | – | 1 (18)  0 | – | – | – | – | – | – |
| 144 months post initiation, *n* (missing)  Yes (%) | – | – | – | – | 1 (18)  0 | – | – | – |
| 156 months post initiation, *n* (missing)  Yes (%) | 1 (18)  1 (5.3) | – | – | – | – | – | 1 (18)  1 (5.3) | – |

Patients at each time point may differ from previous time points. Percentages are calculated with the denominator as the overall population for the cohort (*n* = 19).

### Supplementary Table S8. Clinical outcomes for enlarged tonsils and adenoids, joint stiffness, and carpal tunnel syndrome (primary analysis: Cohort 1 vs Cohort 2)

|  | **Enlarged tonsils and adenoids** | | **Joint stiffness** | | **Carpal tunnel syndrome** | |
| --- | --- | --- | --- | --- | --- | --- |
| **Time of outcome measurement** | **Cohort 1**  ***n* = 15** | **Cohort 2**  ***n* = 15** | **Cohort 1**  ***n* = 15** | **Cohort 2**  ***n* = 15** | **Cohort 1**  ***n* = 15** | **Cohort 2**  ***n* = 15** |
| Index, *n* (missing)  Yes (%) | 15 (0)  0 | 10 (5)  5 (33.3) | 14 (1)  1 (6.7) | 13 (2)  11 (73.3) | 15 (0)  0 | 13 (2)  3 (20.0) |
| 12 months post initiation, *n* (missing)  Yes (%) | – | 1 (14)  1 (6.7) | 2 (13)  2 (13.3) | 1 (14)  1 (6.7) | – | – |
| 24 months post initiation, *n* (missing)  Yes (%) | – | – | 1 (14)  1 (6.7) | 6 (9)  6 (40.0) | – | – |
| 36 months post initiation, *n* (missing)  Yes (%) | – | 1 (14)  1 (6.7) | – | – | – | 1 (14)  1 (6.7) |
| 48 months post initiation, *n* (missing)  Yes (%) | – | 1 (14)  1 (6.7) | 2 (13)  2 (13.3) | – | – | – |
| 60 months post initiation, *n* (missing)  Yes (%) | – | – | 1 (14)  1 (6.7) | 2(13)  2 (13.3) | – | – |
| 72 months post initiation, *n* (missing)  Yes (%) | – | 1 (14)  1 (6.7) | – | – | – | – |
| 84 months post initiation, *n* (missing)  Yes (%) | – | – | – | – | – | 1 (14)  1 (6.7) |
| 96 months post initiation, *n* (missing)  Yes (%) | – | – | – | 2 (13)  2 (13.3) | – | 1 (14)  1 (6.7) |
| 108 months post initiation, *n* (missing)  Yes (%) | – | – | – | 1 (14)  1 (6.7) | – | – |
| 120 months post initiation, *n* (missing)  Yes (%) | – | – | – | 1 (14)  1 (6.7) | – | – |
| 132 months post initiation, *n* (missing)  Yes (%) | – | – | – | – | – | – |
| 144 months post initiation, *n* (missing)  Yes (%) | – | – | – | 1 (14)  1 (6.7) | – | – |
| 156 months post initiation, *n* (missing)  Yes (%) | – | – | 1 (14)  1 (6.7) | – | – | – |

Patients at each time point may differ from previous time points. Percentages are calculated with the denominator as the overall population for the cohort (*n* = 15).

### Supplementary Table S9. Clinical outcomes for enlarged tonsils and adenoids, joint stiffness, and carpal tunnel syndrome (exploratory analysis: Cohort 1 vs Cohort 3)

|  | **Enlarged tonsils and adenoids** | | **Joint stiffness** | | **Carpal tunnel syndrome** | |
| --- | --- | --- | --- | --- | --- | --- |
| **Time of outcome measurement** | **Cohort 1**  ***n* = 11** | **Cohort 3**  ***n* = 11** | **Cohort 1**  ***n* = 11** | **Cohort 3**  ***n* = 11** | **Cohort 1**  ***n* = 11** | **Cohort 3**  ***n* = 11** |
| Index, *n* (missing)  Yes (%) | 11 (0)  0 | 6 (5)  3 (27.3) | 10 (1)  1 (9.1) | 9 (2)  8 (72.7) | 11 (0)  0 | 9 (2)  3 (27.3) |
| 12 months post initiation, *n* (missing)  Yes (%) | – | 1 (10)  1 (9.1) | 1 (10)  1 (9.1) | 1 (10)  1 (9.1) | – | – |
| 24 months post initiation, *n* (missing)  Yes (%) | – | – | 1 (10)  1 (9.1) | 4 (7)  4 (36.4) | – | – |
| 36 months post initiation, *n* (missing)  Yes (%) | – | 1 (10)  1 (9.1) | – | – | – | 1 (10)  1 (9.1) |
| 48 months post initiation, *n* (missing)  Yes (%) | – | 1 (10)  1 (9.1) | 1 (10)  1 (9.1) | – | – | – |
| 60 months post initiation, *n* (missing)  Yes (%) | – | – | 1 (10)  1 (9.1) | 1 (10)  1 (9.1) | – | – |
| 72 months post initiation, *n* (missing)  Yes (%) | – | – | – | – | – | – |
| 84 months post initiation, *n* (missing)  Yes (%) | – | – | – | – | – | – |
| 96 months post initiation, *n* (missing)  Yes (%) | – | – | – | 2 (9)  2 (18.2) | – | – |
| 108 months post initiation, *n* (missing)  Yes (%) | – | – | – | 1 (10)  1 (9.1) | – | – |
| 120 months post initiation, *n* (missing)  Yes (%) | – | – | – | 1 (10)  1 (9.1) | – | – |
| 132 months post initiation, *n* (missing)  Yes (%) | – | – | – | – | – | – |
| 144 months post initiation, *n* (missing)  Yes (%) | – | – | – | 1 (10)  1 (9.1) | – | – |
| 156 months post initiation, *n* (missing)  Yes (%) | – | – | 1 (10)  1 (9.1) | – | – | – |

Patients at each time point may differ from previous time points. Percentages are calculated with the denominator as the overall population for the cohort (*n* = 11).

### Supplementary Table S10. Clinical outcomes for enlarged tonsils and adenoids, joint stiffness, and carpal tunnel syndrome (exploratory analysis: Cohort 4 vs Cohort 5)

|  | **Enlarged tonsils and adenoids** | | **Joint stiffness** | | **Carpal tunnel syndrome** | |
| --- | --- | --- | --- | --- | --- | --- |
| **Time of outcome measurement** | **Cohort 4**  ***n* = 19** | **Cohort 5**  ***n* = 19** | **Cohort 4**  ***n* = 19** | **Cohort 5**  ***n* = 19** | **Cohort 4**  ***n* = 19** | **Cohort 5**  ***n* = 19** |
| Index, *n* (missing)  Yes (%) | 18 (1)  1 (5.3) | 11 (8)  6 (31.6) | 18 (1)  2 (10.5) | 17 (2)  14 (73.7) | 18 (1)  0 | 15 (4)  4 (21.1) |
| 12 months post initiation, *n* (missing)  Yes (%) | – | 1 (18)  1 (5.3) | 2 (17)  2 (10.5) | 1 (18)  1 (5.3) | – | – |
| 24 months post initiation, *n* (missing)  Yes (%) | – | – | 1 (18)  1 (5.3) | 7 (12)  7 (36.8) | – | – |
| 36 months post initiation, *n* (missing)  Yes (%) | – | 1 (18)  1 (5.3) | – | – | – | 1 (18)  1 (5.3) |
| 48 months post initiation, *n* (missing)  Yes (%) | – | 1 (18)  1 (5.3) | 2 (17)  2 (10.5) | – | – | – |
| 60 months post initiation, *n* (missing)  Yes (%) | – | – | 1 (18)  1 (5.3) | 2 (17)  2 (10.5) | – | – |
| 72 months post initiation, *n* (missing)  Yes (%) | – | 1 (18)  1 (5.3) | – | – | – | – |
| 84 months post initiation, *n* (missing)  Yes (%) | – | – | – | – | – | 1 (18)  1 (5.3) |
| 96 months post initiation, *n* (missing)  Yes (%) | – | – | – | 2 (17)  2 (10.5) | – | 1 (18)  1 (5.3) |
| 108 months post initiation, *n* (missing)  Yes (%) | – | – | – | 1 (18)  1 (5.3) | – | – |
| 120 months post initiation, *n* (missing)  Yes (%) | – | – | – | 1 (18)  1 (5.3) | – | – |
| 132 months post initiation, *n* (missing)  Yes (%) | – | – | – | – | – | – |
| 144 months post initiation, *n* (missing)  Yes (%) | – | – | – | 1 (18)  1 (5.3) | – | – |
| 156 months post initiation, *n* (missing)  Yes (%) | – | – | 1 (18)  1 (5.3) | – | – | – |

Patients at each time point may differ from previous time points. Percentages are calculated with the denominator as the overall population for the cohort (*n* = 19).

### Supplementary Table S11. Generalized linear mixed model analyses for the exploratory objectives (Cohort 1 vs Cohort 3 and Cohort 4 vs Cohort 5; reference: older siblings)

|  | **Cohort 1 vs Cohort 3** | | **Cohort 4 vs Cohort 5** | |  |
| --- | --- | --- | --- | --- | --- |
| **Outcome measure** | **Coefficient (95% CI)** | **p value** | **Coefficient (95% CI)** | **p value** | |
| Continuous variables (normal distribution; unity function) |  |  |  |  | |
| Urinary GAG levels,^a^ μg/mg | 177.5 (−305.5, 660.6) | 0.465 | 204.8 (−119.2, 528.8) | 0.212 | |
| Height-for-age Z-score | 1.2 (0.4, 2.0) | **0.003** | 0.8 (0.2, 1.4) | **0.011** | |
| LVMI, g/m^2^ | −50.7 (−84.6, −16.8) | **0.005** | −29.9 (−57.5, − 2.3) | **0.034** | |
| Binary variables (binomial distribution; logit function) |  |  |  |  | |
| Cardiac valve disease | −1.8 (−3.5, −0.1) | **0.042** | −1.3 (−2.7, 0.1) | 0.062 | |
| Normal liver size (palpation) | 8.7 (0.6, 16.8) | **0.035** | 5.3 (2.1, 8.5) | **0.002** | |
| Normal liver size (ultrasound) | 2.8 (−4.4, 10.0) | 0.391 | 1.0 (−2.3, 4.3) | 0.525 | |
| Count variables (Poisson distribution; log function) |  |  |  |  | |
| Enlarged tonsils and adenoids | −2.8 (−4.9, −0.6) | **0.014** | −2.9 (−4.6, −1.2) | **0.001** | |
| Joint stiffness | −2.0 (−4.5, 0.5) | 0.108 | −2.5 (−4.3, −0.7) | **0.007** | |
| Carpal tunnel syndrome | −3.7 (−6.4, −1.0) | **0.008** | −4.0 (−6.4, −1.6) | **0.001** | |
| Surgery | 0.4 (−0.9, 1.6) | 0.564 | −0.1 (−1.1, 0.9) | 0.846 | |

^a^Higher urinary GAG levels were observed in the younger siblings when compared with the older siblings at treatment initiation leading to higher mean urinary GAG levels in Cohort 1 vs Cohort 3 and Cohort 4 vs Cohort 5 in the GLMM analysis.
Strong effects showing differences between sibling cohorts (p < 0.05), which are characterized by a difference in treatment initiation and development of outcomes, are shown in bold font.
CI, confidence internal; GAG, glycosaminoglycan; GLMM, generalized linear mixed model; LVMI, left ventricular mass index.

### Supplementary Fig. S1. Cumulative incidence of first event from birth (exploratory analysis: Cohort 1 vs Cohort 3)

Dashed line shows median age at diagnosis.

CI, confidence interval; HR, hazard ratio.

### Supplementary Fig. S2. Cumulative incidence of first event from birth (exploratory analysis: Cohort 4 vs Cohort 5)

Dashed line shows median age at diagnosis.

CI, confidence interval; HR, hazard ratio.

### Supplementary Fig. S3. Survival for (A) primary analysis of Cohort 1 versus Cohort 2 and exploratory analyses of (B) Cohort 1 versus Cohort 3 and (C) Cohort 4 versus Cohort 5

Dashed line shows median age at diagnosis.

CI, confidence interval; HR, hazard ratio.

### Supplementary Fig. S4. Clinical outcomes for categorical variables (primary analysis: Cohort 1 vs Cohort 2)

C, Cohort.
